# Association between attention deficit hyperactivity disorder and bruxism: a systematic review

**DOI:** 10.3389/fpsyt.2026.1762324

**Published:** 2026-09-01

**Authors:** Anna Carolina Pereira Thimoteo, Manuella S. Coelho, Júlia Meller Dias De Oliveira, Helena Polmann, Cintia Ronchi Lemos, Patrícia Pauletto, Cristine Stefani, Lauren Bohner, Graziela De Luca Canto

**Affiliations:** 1 Universidade Federal de Santa Catarina, Florianópolis, Brazil; 2 Centro Universitario Sociesc, Palhoça, Brazil; 3 Universidad de Las Americas, Quito, Ecuador; 4 Universidade de Brasilia, Brasília, Brazil

**Keywords:** ADHD (attention deficit and hyperactivity disorder), association, bruxism, meta-analysis, systematic review

## Abstract

**Background:**

Attention-deficit/hyperactivity disorder (ADHD) is a prevalent neurodevelopmental condition characterized by symptoms of inattention, disorganization, and/or hyperactivity-impulsivity. Bruxism, defined as repetitive masticatory muscle activity involving clenching or grinding of the teeth and/or forceful mandibular movements, has been associated with several physiological and psychosocial factors. Considering the neurobehavioral profile of individuals with ADHD, a possible association between ADHD and bruxism has been hypothesized.

**Aims:**

To determine whether an association exists between ADHD and bruxism.

**Methods:**

A comprehensive literature search was conducted across five electronic databases and two gray literature sources. Observational studies comparing individuals with ADHD to non-ADHD controls, with data on the presence of bruxism, were included. Independent authors conducted study selection and data extraction. Risk of bias was assessed using the JBI critical appraisal tools. A pairwise meta-analysis was performed using RevMan Web. Odds ratios (ORs) and 95% confidence intervals (CIs) were calculated using a random-effects model. Results: Sixteen studies with 16,348 participants were included. The pooled OR indicated that individuals with ADHD were two times more likely to present bruxism compared to controls (OR 2.16; 95% CI 1.72-2.71; p < 0.00001, I2 51%). Subgroup analyses by age group showed a consistent association across children, adolescents, and adults. Moderate heterogeneity and potential small-study bias (with little impact on the pooled results after sensitivity analysis) were observed.

**Conclusions:**

Current evidence supports a possible association between ADHD and bruxism.

## 1 Introduction

Attention-Deficit/Hyperactivity Disorder (ADHD) is a neurodevelopmental disorder marked by impairing levels of inattention, disorganization, and/or hyperactivity-impulsivity. Inattention and disorganization entail inability to stay on task, seeming not to listen, and losing materials necessary for tasks, at levels that are inconsistent with age or developmental level (DSM). It is most diagnosed in childhood and tends to decrease in prevalence with age (1). Globally, the prevalence of ADHD is estimated at 7.3%, with approximately 2.5% of cases occurring in adults (1).

Currently, the diagnosis of ADHD requires a clinical interview to assess the core symptoms and related functional impairments, as outlined in the fifth edition of the Diagnostic and Statistical Manual of Mental Disorders(DSM-5) (2). Although ADHD has a significant genetic basis, environmental factors also play a role in its development, many of which remain incompletely understood (3). Extensive evidence from epidemiological, clinical, and longitudinal studies has consistently demonstrated the persistence of ADHD symptoms, namely inattention, hyperactivity, and impulsivity, and their substantial impact (3). These include academic and occupational difficulties, impaired social functioning, and an increased risk of accidents (3). In addition, common psychiatric comorbidities, such as anxiety and mood disorders, are frequently observed in individuals with ADHD (3).

A potential association between ADHD and bruxism was first suggested by Malki et al. in 2005 (4). Bruxism is a repetitive jaw-muscle activity involving teeth grinding or clenching, and/or mandibular bracing or thrusting (5). Bruxism has a multifactorial etiology, influenced by psychosocial, physiological, and behavioral factors including respiratory issues, attention deficits, depression, stress, anxiety, and tension (6–8). Bruxism manifests in two distinct circadian patterns: during sleep (sleep bruxism) and during wakefulness (awake bruxism) (5).

The assessment of bruxism is complex and can be categorized into three main approaches: self-report (subject-based), clinical examination (clinically based), and device-based methods such as electromyography and polysomnography (PSG) (5). The reported prevalence of sleep bruxism (SB) in adults varies depending on the assessment method: approximately 12.8% (± 3.1%) when based on self-reports (9), and 7.4% when measured using PSG (10). Furthermore, SB tends to decline with age, affecting about 14% of children, 8% of adults, and just 3% of individuals over 60 years old (6). Prevalence rates for awake bruxism (AB) are reported to range from 16% to 32% (11).

A previous systematic review (12) found a higher prevalence of both sleep and AB among children and adolescents with ADHD, compared to those without the disorder. However, this review was limited in scope to pediatric populations and included studies up to April 2019. Therefore, a new systematic review was conducted, broadening the inclusion criteria to encompass individuals of all ages to answer the research question: “Is there an association between ADHD and bruxism?

## 2 Methods

### 2.1 Eligibility criteria

The research question was developed based on the PECOS acronym (**Box 1**).

Box 1 **PECOS acronym description.**

**Table:** 

P (Population)	Children, adolescents, and adults
E (Exposure)	Attention Deficit Hyperactivity Disorder (ADHD)
C (Comparison)	Individuals without ADHD
O (Outcomes)	Occurrence of bruxism (Sleep Bruxism or Awake Bruxism)
S (Type of study)	Observational studies

#### 2.1.2 Inclusion criteria

Observational studies comparing the occurrence of bruxism - whether AB, SB, or both - between individuals with and without ADHD were included. Studies conducted in various settings, including hospitals, clinics, sleep laboratories, or community-based populations, were considered eligible. The study population consisted of children (up to 12 years old), adolescents (13 to 18 years old), and adults (over 18 years old), regardless of sex, race, ethnicity, or socioeconomic background. ADHD must have been diagnosed based on any edition of the *Diagnostic and Statistical Manual of Mental Disorders* (DSM) (2, 13).

The presence of bruxism must have been identified according to the criteria established by the International Consensus (14), using one or more of the following assessment methods: subject-based (self-report), clinically based (clinical examination), or device-based tools (e.g., electromyography, polysomnography).

#### 2.1.3 Exclusion criteria

The following studies were excluded:

1. Studies involved specific populations, such as individuals with congenital anomalies; a history of bipolar disorder, psychosis, anxiety disorders, Tourette’s disorder, autism; a history of substance abuse; or other neurological conditions associated with significant attention impairment;
2. Studies that did not use any edition of the *Diagnostic and Statistical Manual of Mental Disorders* (DSM) for the diagnosis of ADHD;
3. Studies that did not specify the method used to detect bruxism and/or ADHD; lacked data on bruxism; did not include a control group; or did not assess the association between bruxism and ADHD;
4. Review articles, letters, editorials, books, conference abstracts, case reports, case series, opinion pieces, or clinical guidelines;
5. Studies for which the full text was not available, even after three contact attempts with the corresponding authors over a three-week period.

### 2.2 Information sources and search strategy

A preliminary search strategy using terms related to “bruxism” and “attention deficit disorder” was formulated and refined with the assistance of a health sciences librarian. Subsequently, a comprehensive search strategy was developed for each database (**Supplementary Appendix A1**). A systematic search was conducted in the following databases from inception to the present: Embase, LILACS (via BVS), MEDLINE (via PubMed), Scopus, and Web of Science. Gray literature was explored through Google Scholar and ProQuest Dissertations & Theses Index. The search was conducted on June 5, 2025. The reference lists of included studies were reviewed, and input from field experts was sought, specifically from authors of four or more studies included in this review. Studies from a previous systematic review (12) were also assessed to determine whether they met the eligibility criteria.

### 2.3 Data management

The files from each database were imported to EndNote(EndNote-Web™, Clarivate™, Jersey, USA) (15). They were merged, and duplicate references were identified and excluded. After that, a single file was imported into the Rayyan^®^ online application (Qatar Computing Research Institute, Data Analytics, Doha, Qatar) (16),where the selection process based on the eligibility criteria was carried out independently by three authors (ACPT, MSC, JMDO).

### 2.4 Selection process

First, three authors (ACPT, MSC, JMDO) read the abstracts of five studies and performed a preliminary pilot test according to the eligibility criteria to calibrate the process. After the pilot test, a two-phase process was executed by the same three independent authors (ACPT, MSC, JMDO). In phase 1, they screened the titles and abstracts of all identified references. Studies failing to meet the eligibility criteria were excluded. During phase 2, the same three authors applied the eligibility criteria to the full texts of the studies. In cases of disagreement concerning article inclusion or exclusion, a fourth author (HP) was consulted, and consensus was reached through discussion.

### 2.5 Data collection process

An initial data extraction template was created using Microsoft Excel version 4.7.7 (Microsoft Office 365, Microsoft^®^ Corporation, Redmond, Washington, USA) (17). The variables collected focused on capturing specific details related to the PECOS framework.

Data extraction from the included studies was performed by one author (ACPT) and cross-checked by another author (JMDO) using the data extraction tool developed by the authors. Discrepancies were addressed through discussion between the two authors. If disagreements persisted, a third author (HP) was consulted. In cases involving duplicate publications or primary studies with multiple reports, all available data were collected and incorporated into a comprehensive dataset aggregating information from all known publications. The corresponding authors of the original studies were contacted to request any missing data. This was achieved by sending up to three emails, with an interval of 15 days between each one. If the data could not be obtained, the studies were considered incomplete, and only available data were analyzed.

### 2.6 Data items

The following data were collected from the included studies: study characteristics (author, year, country, study design, setting); objectives, participant characteristics (population, age, sex - % male/female); criteria for ADHD diagnosis; number of patients with ADHD; number of patients without ADHD; criteria for bruxism diagnosis; number of patients with bruxism; number of patients without bruxism; type of bruxism (sleep/awake, primary/secondary); number of bruxism events in both groups; results; conclusions; funding; and conflicts of interest.

### 2.7 Risk of bias in individual studies

The risk of bias in the included studies was independently assessed by two authors (ACPT, JMDO) using JBI’s critical appraisal tools specific to each study design (18). Before applying the tool, the authors deliberated on the choice of tool and established evaluation parameters. Subsequently, a calibration exercise was conducted between the evaluators by using two included articles. Any discrepancies between the authors were resolved through discussion and consultation with a third author (HP). Figures regarding the risk of bias were created using the online tool *robvi*s (Risk-Of-Bias VISualization) (National Institute for Health Research) (19).

### 2.8 Effect measures and data synthesis

A narrative synthesis was conducted to summarize the results of each included study. Pairwise meta-analyses of associations were performed using RevMan Web (Review Manager Web, available at https://revman.cochrane.org/, accessed in September, 2025). The considered measure of association was odds ratios (OR) with 95% confidence intervals (95% CI). The inverse-variance method under a random-effects model was used for both overall and subgroup analyses.

Statistical heterogeneity was evaluated among the results of different trials using the chi-square (Chi^2^) test, with significance defined as P<0.1. The I^2^ statistic was utilized to assess heterogeneity within studies in each analysis. Evidence of heterogeneity was considered when the Chi² test´s *p*-value was < 0.10 and the I² value was 50% or higher. In that case, possible reasons for heterogeneity were explored through subgroup analyses. The influence of risk of bias on the overall effect estimate was investigated by excluding studies with a high risk of bias from each meta-analysis (sensitivity analysis) (20).

### 2.9 Subgroup analysis

Subgroup analyses were planned according to age group (children, adolescents, or adults) and bruxism type, categorized as sleep bruxism (SB) or awake bruxism (AB), and by assessment method (self-reported, clinically diagnosed, or identified through device-based tools).

### 2.10 Sensitivity analysis

A sensitivity analysis was performed to explore the impact of studies at high risk of bias and studies with small sample (N < 100) on the estimate of the overall effect, by removing studies with these characteristics from the meta-analysis.

### 2.11 Assessment of reporting bias

Publication bias was assessed through funnel plots and Egger’s test (21).

### 2.12 Confidence in cumulative evidence

Given the lack of guidance from the GRADE Working Group for systematic reviews of association and considering that the “Requirements for claiming the use of GRADE” advise against modifications and adaptations of the GRADE approach (22), the certainty of the evidence was not assessed.

## 3 Results

### 3.1 Study selection

A total of 900 records were identified through searches of electronic databases and gray literature sources. After the removal of duplicates, 554 records remained and were screened by title and abstract in phase-1. Of these, 69 records were selected for full-text screening in phase-2. During this phase, the full texts were assessed for eligibility, resulting in the inclusion of 16 studies that met the predefined inclusion criteria (4, 23–37).

No author was classified as an expert contributor, as none had authored four or more of the included studies. Therefore, no additional eligible studies were identified through expert consultation. Reference list screening added no additional studies either.

**Figure 1:**
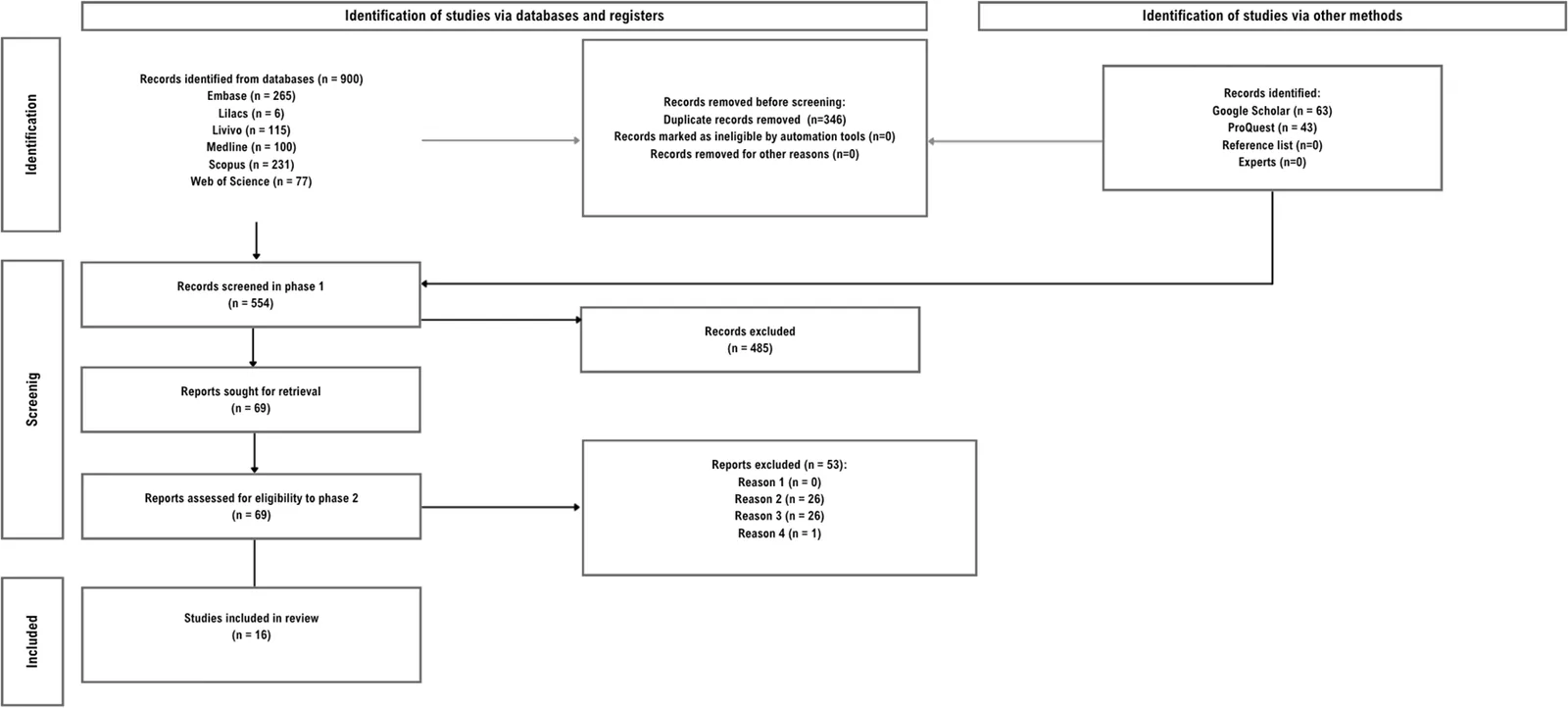
Flow diagram of literature searches and selection criteria (adapted from preferred reporting items for systematic reviews and meta‐analysis).

The study selection process is illustrated in the PRISMA flow diagram (**Figure 1**). A list of excluded full-text articles, along with reasons for exclusion, is provided in **Supplementary Appendix A2**.

### 3.2 Study characteristics

The included studies were conducted across four continents: Asia (26, 27, 29, 30, 34, 36), America (4, 24, 25, 31–33), Europe (23, 28, 35, 37). The publication date ranged from 2005 Malki (4) to 2024 Alessandri-Bonetti (23); Malacarne (31).

Of the 16 studies included, three focused exclusively on children (aged 6–12 years) (29, 32, 35), twelve examined populations consisting of children and adolescents (aged 4–18 years) (4, 23–28, 30, 33, 34, 36, 37), and only one study investigated adults (aged 18–50 years) (31). All included studies adopted a cross-sectional design.

### 3.3 Risk of bias

**Figure 2:**
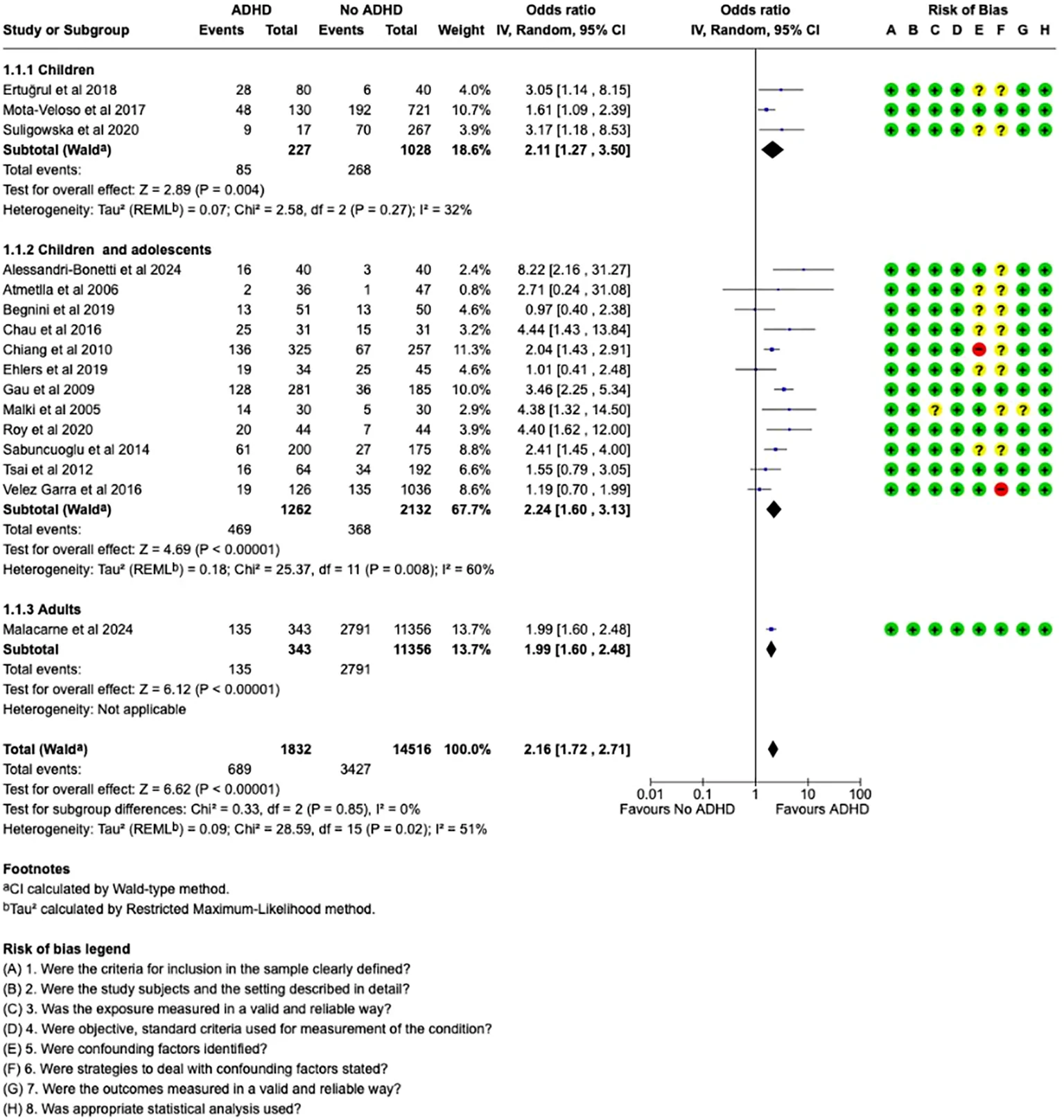
Forest plot of the subgroup meta-analyses by age group comparing the presence of bruxism in individuals with and without ADHD (n=16).

Based on the JBI critical appraisal tool (18) (**Supplementary Appendix A3**), five studies (30–33, 36), were classified as having a low risk of bias, nine studies (4, 23–26, 28, 29, 34, 35) as having a moderate risk, and two studies (27, 37), were rated as having a high risk of bias across all assessed domains. Risk of bias assessment indicated that most studies had a low risk of bias in the majority of domains. Nonetheless, some studies have raised concerns, particularly in JBI checklist items D, E, and F (related to confounding factors), and H (statistical analysis) (**Figure 2**).

### 3.4 Results of individual studies

The results of individual studies are presented on **Table 1**.

**Table 1:** Methodological characteristics and main findings of the included studies investigating the association between Attention-Deficit/Hyperactivity Disorder (ADHD) and bruxism.

Author, year, country	Study design	Setting	Objetives	Population	Age Range (years)	Sex (M-F%)	Criteria for ADHD diagnosis	Pacients with ADHD (n)	Pacients without ADHD (n)	Criteria for bruxism detection	Bruxism + ADHD (n)	No Bruxism + ADHD (n)	Bruxism + Control (n)	No Bruxism + Control (n)	Results	Conclusions	Funding	Conflict of interest
Alessandri-Bonetti et al., 2024, Italy (23)	Cross-sectional	Paediatric Dentistry Unit of Fondazione Policlinico Universitario, Italy	To investigate the prevalence of OSA risk in children with ADHD; to observe the prevalence of malocclusions and SB, and examine associations with OSA	Children and adolescents	6 - 16	77.5 / 22.5	DSM-V	40	40	Parent-reported and clinical examination	16	24	3	37	A significantly higher prevalence of probable SB was observed among children with ADHD, with 40% (16 out of 40) of the participants in this group exhibiting the condition. In contrast, only 7.5% (3 out of 40) of the children in the control group presented with bruxism. This difference was statistically significant (p = .001)	Children with ADHD are more likely to have probable SB and a higher risk of OSA, but malocclusion patterns do not differ significantly from those of healthy children.	NR	Authors declared no conflicts of interest.
Atmetlla et al., 2006, Colombia (24)	Cross-sectional	Pontificia Universidad Javeriana and the Military Hospital in Bogotá, Colombia	To evaluate orofacial and behavioral characteristics of children with ADHD during dental visits	Children and adolescents	5 -13	83.3 / 16.7	DSM-IV	36	47	Parent-reported and clinical examination	2	34	1	46	Significantly higher rates of oral habits (nail-biting, lip biting, bruxism) in the ADHD group ADHD group showed more orofacial alterations (fissured tongue, saburral tongue, hypomineralization, pathological wear facets) SNAP-IV and ADHDT scores significantly higher in ADHD group for inattention, hyperactivity, and impulsivity No significant differences in molar relation, overjet/overbite, or profile	Children with ADHD had more oral habits (including bruxism), and specific orofacial characteristics compared to controls. ADHD-related behavioral traits impacted dental visit behavior. The study supports the use of SNAP-IV and ADHDT in dental settings to help identify and manage ADHD patients effectively.	NR	Authors declared no conflicts of interest.
Brancher et al., 2019, Brazil (25)	Cross-sectional	Universidade Positivo, Brazil	To evaluate the oral health conditions of children and adolescents with ADHD and compare them with a control group	Children and adolescents	9 -13	67.0 / 33.0	DSM-IV	51	50	Parent-reported and clinical examination	13	38	13	37	Bruxism occurred in 25% of ADHD group and 26% of controls (p = 0.953); Bruxism: No significant difference between groups (p = 0.953)	Children and adolescents with ADHD presented poorer oral health (higher plaque and gingival bleeding indexes). Parental supervision and use of medication influenced bruxism frequency. Dental professionals should pay special attention to this population.	NR	NR
Chau et al., 2016, China (26)	Cross-sectional	University-affiliated dental hospital, China	To assess and compare oral health status and oral-health behaviors among children with and without ADHD	Children and adolescents	12 -18	87.1 / 12.9	DSM-IV	31	31	Parent-reported	25	6	15	16	Children with ADHD had higher rates of bruxism, dislike of dentists, and reduced toothbrushing times compared to the control group. There were no significant differences in dental caries or tooth wear, but children with ADHD had more gingival bleeding and attended government dental clinics more frequently	The study found that children with ADHD have poorer oral hygiene and higher bruxism rates, emphasizing the need for targeted oral health interventions.	NR	NR
Chiang et al., 2010, China (27)	Cross-sectional	National Taiwan University Hospital and associated institutions, Taiwan	To investigate the association between ADHD symptoms and subtypes with sleep schedules, daytime napping, and sleep disorders in children and adolescents	Children and adolescents	10–17	70.9 / 29.1	DSM-IV + K-SADS-E	325	257	Based on K-SADS-E interviews and confirmed by parent reports of teeth grinding	136	189	67	190	ADHD subtypes were linked with various sleep disorders, such as bruxism, insomnia, and sleep terrors. ADHD-C was particularly associated with a higher prevalence of sleep disorders, while ADHD-I was linked to hypersomnia. ADHD symptoms were correlated with earlier bedtime, later rise times, and longer sleep duration on schooldays	There are significant associations between ADHD subtypes and sleep disorders. The study highlights the need to consider ADHD symptoms and subtypes in assessing and managing sleep disorders in clinical settings.	NR	NR
Ehlers et al., 2019, Germany (28)	Cross-sectional	Residential Care Facilities in Rural Rhineland-Palatinate, Germany	To evaluate the oral health parameters of children and adolescents with and without ADHD living in residential care settings	Children and adolescents	9 -15	74.2 / 25.8	DSM V and IC-10	34	45	Cinical examination	19	15	25	20	No significant differences were found between the ADHD and control groups in terms of oral health parameters, such as DMFS/DMFT scores, approximal plaque index (API), or bruxism prevalence. The ADHD group tended to have higher DMFS/DMFT values and consumed more sugary drinks and snacks. Orthodontic treatment was less frequent in the ADHD group compared to the control group	The study found no significant difference in oral health between children with and without ADHD living in residential care. However, there were trends indicating poorer oral hygiene and higher DMFT values in the ADHD group, emphasizing the need for better oral health supervision and dietary guidance.	NR	NR
Ertuğrul et al., 2018, Turkey (29)	Cross-sectional	Department of Pediatric Dentistry, Pamukkale University, Turkey	To evaluate the impact of psychostimulant therapy on oral health and saliva characteristics in children with ADHD	Children	7 -12	82.5 / 17.5	DSM-IV	80	40	Parent-reported and clinical examination	28	52	6	34	Children with ADHD, both medicated and non-medicated, had higher rates of bruxism and dental erosion compared to the control group, though the differences were not statistically significant. Dry mouth and lower salivary flow rates were more common in children on medication	Psychostimulant therapy does not significantly alter saliva properties but may be associated with dry mouth and increased fluid consumption. Regular dental follow-ups and oral hygiene practices are recommended for children with ADHD.	NR	NR
Gau et al., 2009, China (30)	Cross-sectional	National Taiwan University Hospital, Taiwan	To investigate the sleep schedules, problems, and disorders among adolescents with persistent ADHD and those with subthreshold ADHD symptoms	Children and adolescents	11 -17	81.1 / 18.9	DSM-IV + K-SADS-E	281	185	Presence of teeth grinding during sleep, reported during psychiatric interviews	128	153	36	149	Adolescents with persistent and subthreshold ADHD were more likely to have sleep disorders, such as insomnia, sleep terrors, and bruxism, compared to controls. The presence of psychiatric comorbidities increased the risk of sleep disorders	ADHD is associated with a higher prevalence of sleep problems and disorders, even when accounting for comorbid psychiatric conditions. The study emphasizes the importance of screening for sleep problems in ADHD patients.	National Health Research Institute, Taiwan	The authors disclosed no financial conflicts, though Dr. Gau had received research support from Janssen-Cilag and Eli Lilly​
Malacarne et al., 2024, United States (31)	Cross-sectional	Tufts University School of Dental Medicine, United States	To assess whether common orofacial pain complaints such as jaw pain, jaw clicking, teeth clenching, and headaches are more prevalent in dental patients with an ADHD diagnosis and/or using psychostimulant medications	Adults	18 - 50	46.2 / 53.8	DSM-V	343	11,356	Self-report only	135	208	2791	8565	Patients with ADHD and on stimulants reported significantly higher odds of clenching, jaw pain, and headaches compared to non-ADHD, non-stimulant users.Patients taking stimulants (without ADHD diagnosis) also had higher odds of clenching and headaches	There is an association between ADHD diagnosis, psychostimulant use, and orofacial pain symptoms. Stimulant use, even in non-ADHD patients, is linked to symptoms like clenching and headaches	NR	Authors declared no conflicts of interest.
Malki et al., 2005, United States (4)	Cross-sectional	Franciscan Children’s Hospital Dental Clinic, United States, and King Fahad Hospital, Jeddah, Saudi Arabia	To evaluate the prevalence of bruxism in children diagnosed with ADHD and examine the influence of ADHD medication on bruxism severity	Children and adolescents	5 - 15	80.0 / 20.0	Medical history and confirmed by a physician	30	30	Parent-reported and clinical examination	14	16	5	25	Children with ADHD had a significantly higher prevalence of bruxism compared to controls, particularly those on CNS-stimulant medications. ADHD children taking medication had more worn teeth compared to those not on medication	There is a strong association between ADHD medication use, particularly CNS stimulants, and increased prevalence of bruxism. The findings suggest that ADHD children should be regularly monitored for bruxism, especially those taking medications.	NR	NR
Mota-Veloso et al., 2017, Brasil (32)	Cross-sectional	Schools in Diamantina, Brazil	To evaluate the effects of signs of ADHD and SES on SB and tooth wear among schoolchildren	Children	6 -12	45.2 / 54.8	DSM-IV	130	721	Parent-reported and clinical examination	48	82	192	529	ADHD signs had a moderate effect on SB, and socio-economic status had both direct and indirect effects on tooth wear and bruxism. Socio-economic status influenced bruxism indirectly through sucking habits	ADHD signs and socio-economic status have complex effects on SB and tooth wear, underlining the need for multidisciplinary approaches in treating affected children.	The Brazilian Coordination of Higher Education (CAPES), the Research Foundation of the State of Minas Gerais (FAPEMIG), and the National Council for Scientific and Technological Development (CNPq), Brazil	NR
Roy et al., 2020, Canadá (33)	Cross-sectional	Orthodontic Clinic, Université de Montréal, and Division of Dentistry, Montreal Children’s Hospital, Canadá	To assess the severity of dental malocclusion in children with ADHD and its association with parafunctional oral habits such as bruxism	Children and adolescents	6 - 17	70.5 / 29.5	DSM V	44	44	Parent-reported	20	24	7	37	Children with ADHD had a significantly higher severity of dental malocclusion, more dental rotations, and a higher prevalence of parafunctional oral habits, including bruxism	Children with ADHD are at increased risk for parafunctional oral habits and dental malocclusion, highlighting the need for preventive and therapeutic orthodontic strategies.	NR	NR
Sabuncuoglu et al., 2014, Turkey (34)	Cross-sectional	Marmara University Hospital, Turkey	To investigate the association between breastfeeding duration and parafunctional oral habits, such as bruxism, in children with and without ADHD	Children and adolescents	7 - 17	75.0 / 25.0	DSM-IV	200	175	Parent-reported	61	139	27	148	Children with ADHD had significantly shorter exclusive breastfeeding duration and a higher prevalence of parafunctional oral habits, including bruxism, compared to the control group	The findings suggest that insufficient exclusive breastfeeding may be linked to an increased risk of ADHD and related parafunctional oral habits.	NR	NR
Suligowska et al., 2021, Poland (35)	Cross-sectional	Medical University of Gdańsk and Medical University of Warsaw, Poland	To compare the prevalence of parafunctions, signs, and symptoms of TMD in children with and without ADHD	Children	10–12	73.7 / 26.3	DSM- IV	17	267	Child self-report via structured clinical interview	9	8	70	197	Children with ADHD exhibited significantly higher rates of parafunctions, including bruxism, gum chewing, and nail biting, compared to the control group. They also had a higher prevalence of TMD signs and symptoms	Children with ADHD are at greater risk for developing parafunctions and TMD. Interdisciplinary treatment involving both psychiatrists and dentists is recommended.	City of Sopot, Medical University of Gdańsk, Servier Poland, and the Young Scientist program	NR
Tsai et al., 2012, China (36)	Case-control	National Taiwan University Hospital, Taiwan	To compare sleep problems in children with ASD, ADHD, epilepsy, and typically developing children	Children and adolescents	6 - 16	60.9 / 39.1	DSM-IV	64	192	Parent-reported	16	48	34	158	Children with ADHD had significantly higher rates of sleep disturbances, including bruxism and restless legs syndrome, compared to typically developing children. No significant differences in sleep issues were found between children with ADHD and those with ASD	Sleep disturbances are more common in children with ADHD and ASD compared to typically developing children. The study emphasizes the importance of assessing and managing sleep problems in clinical practice for children with neurodevelopmental disorders.	National Science Council, Taiwan	NR
Velez-Galarraga al., 2016, Spain (37)	Cross-sectional	Pediatric Neurology Unit, Clínica Universidad de Navarra, Spain	To determine the prevalence of sleep disorders in children with ADHD and in a control population, and to examine the relationship between sleep disorders and ADHD symptoms	Children and adolescents	5 -18	43.4 / 56.6	DSM-IV	126	1036	Based on the PSQ, focusing on parent-reported rhythmic movements and bruxism	19	107	135	901	Children with ADHD showed a higher prevalence of bedtime resistance, difficulty falling asleep, and rhythmic movements. Sleep issues were more prominent in untreated ADHD cases, and children under 12 exhibited more sleep disturbances compared to older children	Sleep disturbances are common in children with ADHD, especially in untreated cases. The study suggests a need for interventions to improve sleep quality to manage ADHD symptoms effectively.	NR	NR

ADHD, Attention-Deficit/Hyperactivity Disorder; ADHDT, Attention-Deficit/Hyperactivity Disorder Test; ASD, Autism Spectrum Disorders; CAPES, Coordenação de Aperfeiçoamento de Pessoal de Nível Superior; CNPq, Conselho Nacional de Desenvolvimento Científico e Tecnológico; DMFS, Decayed, Missing or Filled Surfaces; DMFT, Decayed, Missing, and Filled Teeth; DSM-IV, Diagnostic and Statistical Manual of Mental Disorders, Fourth Edition; DSM-V, Diagnostic and Statistical Manual of Mental Disorders, Fifth Edition; FAPEMIG, Fundação de Amparo à Pesquisa do Estado de Minas Gerais; F90.0, Attention-deficit hyperactivity disorder, predominantly inattentive type; F90.1, Attention-deficit hyperactivity disorder (ADHD), predominantly hyperactive type; F90.8, Other hyperkinetic disorders; GBI, Gingival Bleeding Index; ICD-10, International Statistical Classification of Diseases and Related Health Problems, 10th Revision; K-SADS-E, Kiddie Schedule for Affective Disorders and Schizophrenia – Epidemiologic version; K-SADS-PL, Schedule for Affective Disorders and Schizophrenia for School-Age Children-Present and Lifetime Version; NIMH, National Institute of Mental Health; NR, Not Reported; OSA, Obstructive Sleep Apnea; PSQ, Pediatric Sleep Questionnaire; PTDs, Persistent Tic Disorders; SB, Sleep Bruxism; SES, Socio-Economic Status; SNAP-IV, Swanson, Nolan, and Pelham, Version IV; TMD, Temporomandibular Disorders; VPI, Visible Plaque Index.

### 3.5 Synthesis of results

#### 3.5.1 Meta-analysis

A total of 16 studies were included in the meta-analysis to compare the occurrence of bruxism between individuals with and without ADHD. Altogether, the data comprised 16,348 participants (1,832 with ADHD and 14,516 without ADHD). The pooled analysis showed that individuals with ADHD had a significantly higher chance of presenting bruxism compared to controls, with an OR of 2.16 (95% CI 1.72 to 2.71; P<0.00001, I^2^ = 51%). This indicates that the odds of presenting bruxism were 2.16 times higher in individuals with ADHD compared to those without.

#### 3.5.2 Subgroup analyses

While the protocol initially planned subgroups based on age (children, adolescents, or adults), type of bruxism (sleep/awake and diagnostic certainty), ADHD diagnosis method, and sampling strategy (convenience vs. population-based), only subgrouping by age group was possible. Subgroup analyses (**Figure 2**) were conducted by age group. The OR was 2.11 (95% CI 1.27 to 3.50; P = 0.004; I² = 32%) for children, 2.24 (95% CI 1.60 to 3.13; P < 0.00001; I² = 60%) for children and adolescents, and 1.99 (95% CI 1.60 to 2.48; P < 0.00001; I² was not calculated, since only one study was included in this subgroup) for adults. The test for subgroup differences was not statistically significant (I² = 0%), indicating a consistent direction of effect across age groups. Overall heterogeneity was moderate (I² = 51%), suggesting some variability between studies that could not be attributed solely to sampling error.

#### 3.5.3 Sensitivity analysis

Sensitivity analysis was conducted to evaluate the impact of the risk of bias on the results by removing studies with a high risk of bias from the meta-analysis (27, 37). The results showed a low impact of these studies on the pooled results (OR 2.32; 95% CI 1.79 to 3.00; P < 0.00001; I^2^ = 48%) after removing studies with a high risk of bias, compared with the overall estimate (OR 2.16; 95% CI 1.72 to 2.71; P < 0.00001, I^2^ = 51%). Additionally, the impact of small studies was evaluated by excluding those with fewer than 100 participants. Again, sensitivity analysis revealed a small impact of small-sample studies on the pooled results (OR 1.98; 95% CI 1.60 to 2.46), compared to the overall OR of 2.16 (95% CI 1.72 to 2.71; P < 0.00001, I^2^ = 51%). The subgroup “Children and Adolescents” was the most affected (after removing studies with a small sample size OR 1.89; 95%CI 1.32 to 2.71; P = 0.0005; I^2^ = 65%, compared to the original OR 2.24; 95%CI 1.60 to 3.13, P < 0.00001; I^2^ = 60%). Forest plots presenting the sensitivity analyses can be found in **Figure 2**.

### 3.6 Assessment of reporting bias

**Figure 3:**
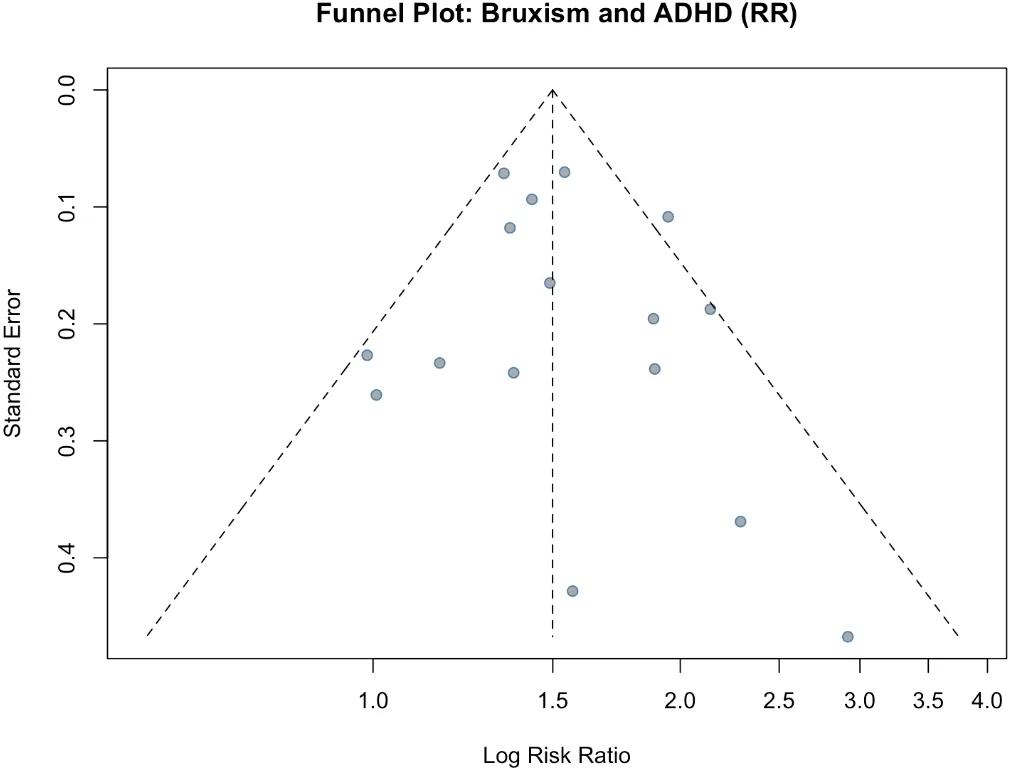
Funnel plot assessing publication bias for the meta-analysis.

Visual inspection of the funnel plot revealed moderate asymmetry, suggesting potential publication bias (**Figure 3**). The plot shows an overrepresentation of smaller studies (with higher standard errors) reporting stronger associations, predominantly positioned on the right side of the funnel, suggesting a skew toward positive findings. This pattern may reflect a tendency toward the publication of studies with significant positive associations between ADHD and bruxism, which could overestimate the pooled effect size. This interpretation is further supported by Egger’s regression test (p = 0.0015), which confirmed statistically significant small-study effects, consistent with the observed asymmetry. Yet, sensitivity analysis after removing small-sample studies revealed a low impact on the pooled results of the meta-analysis (see item 3.5.3 - Sensitivity analysis).

## 4 Discussion

This systematic review aimed to investigate whether there is a possible association between ADHD and bruxism. The topic has aroused interest in the literature due to the increasing incidence of both conditions worldwide, in different age groups (1, 6, 9–11), Although the hypothesis of an association between bruxism and ADHD was raised by Malki et al. (4) more than 20 years ago, the possible association between the conditions is not well established in the literature.

According to the meta-analysis results, individuals with ADHD are two times more likely to have bruxism compared to controls. These findings are consistent with those of a previous systematic review published in 2020, which found that children and adolescents diagnosed with ADHD had a 2.94 times higher chance of presenting sleep bruxism and awake bruxism compared to controls without ADHD (12).

The possible association between ADHD and bruxism can be attributed to several factors. Individuals with bruxism were found to have higher levels of some self-reported stress symptoms as assessed through questionnaires. Stress biomarkers like epinephrine, norepinephrine, cortisol, adrenaline, dopamine, noradrenaline and prolidase enzyme levels also showed a positive association with SB (8). Also some specific symptoms of the anxiety disorders spectrum might be associated with SB (38). A recent review has highlighted the role of stress both as a precipitating and an initiating factor in the genesis of bruxism. The review indicated that chronic stress can degenerate the hippocampus and destabilize the mesocortical dopaminergic pathway, which is responsible for the control of involuntary muscle movements (39). In the same way, individuals with ADHD often have higher levels of stress, anxiety, hyperactivity, and impulsivity (40), which may contribute to the development of bruxism.

From a neurobiological perspective, both ADHD and bruxism may share mechanisms involving dysregulation of dopaminergic pathways, altered arousal responses during sleep, and dysfunction in inhibitory motor control (6, 40), ADHD is associated with hypoactivity of dopamine transmission in specific brain regions, whereas bruxism has been linked to transient hyperactivity in the dopaminergic system during sleep arousals. Shared genetic factors and altered regulation of the hypothalamic-pituitary-adrenal (HPA) axis, influencing stress reactivity, may also play a role (41).

The literature suggests that the pathophysiology of bruxism may involve peripheral and central mechanisms (39). Bruxism may be associated with transient arousals during non-rapid eye movement (non-REM) sleep, during which excitatory neurotransmitters momentarily overcome inhibitory mechanisms, resulting in the activation of the jaw muscles (6). Furthermore, it is known that bruxism may be associated with psychosocial factors, stress, anxiety, and genetic predisposition (42). Other associated factors are the continuous use of medications, such as duloxetine, paroxetine, venlafaxine, barbiturates, and methylphenidate, which may be associated with SB (43), and the abuse of drugs, caffeine, smoking, and alcohol consumption (44).

Furthermore, it is important to highlight that clinical guidelines recommend stimulants as the first-choice medication for ADHD due to their high efficacy (1, 45), such as amphetamines or methylphenidate. These medications have shown a strong association with signs and symptoms of secondary bruxism (4). Malki et al. (4) found a strong association between the use of ADHD medications specifically central nervous system (CNS) stimulants such as methylphenidate and amphetamines and the presence of bruxism in individuals with ADHD, with a significantly higher prevalence of bruxism compared to controls, particularly among those taking CNS stimulant medications.

Even though ADHD has long been considered a childhood disorder, more recent studies establish that the harmful symptoms of ADHD persist into adulthood in a considerable portion of diagnosed children and adolescents (about 65%) (1, 45), It is important to highlight that the prevalence of both conditions decrease with age. The prevalence of ADHD is approximately 5% in children (45) and 2.5% in adults (1). The prevalence of SB, on the other hand, decreases over time, being 14% in children, 8% in adults, and 3% in patients over 60 years of age (6). This might be the reason why, although our systematic review did not restrict the age of participants, we found only one study in adults investigating the association between ADHD and bruxism (31). Moreover, this adult study had the largest sample: of the 16,000 participants included in our systematic review, more than 11,000 were from this study (31). Therefore, its precision is considerably higher (much greater than all the other studies combined in the meta-analysis), which increases the confidence that the results are close to the true effect.

One important aspect to consider is the variability in the diagnostic criteria for bruxism. It is known that objective assessments, such as PSG or electromyography, provide a conclusive diagnosis of bruxism (5). Still, they are less frequently used compared to subjective tools, such as questionnaires or parental reports. Such methodological differences can impact the accuracy and comparability of the diagnosis, potentially influencing the measured association with bruxism.

The heterogeneity observed in our meta-analysis can be attributed to differences in sample characteristics (age range, cultural background, socioeconomic status), and diagnostic criteria for bruxism. Although random effects models were used to account for variability, the magnitude of heterogeneity underscores the need for standardized diagnostic protocols in future studies.

Clinically, the findings of this study highlight the importance of interdisciplinary diagnostic strategies that involve dental and mental health professionals. Dentists should remain vigilant for behavioral patterns and clinical signs suggestive of ADHD, particularly in patients with bruxism or other parafunctional habits, and consider referral for psychological or psychiatric evaluation when appropriate. Conversely, psychiatrists, psychologists, and other mental health professionals should seek training to recognize orofacial complaints such as jaw pain, tooth wear, or headaches in patients diagnosed with ADHD, investigating a potential association with bruxism. This multidisciplinary awareness can promote early detection, optimize management, and ultimately improve outcomes through coordinated care, improving the quality of life for patients. The discussion about the relationship between ADHD and bruxism will benefit both health professionals and patients, as well as policymakers, allowing them to make informed decisions regarding this association.

Although this systematic review found evidence of an association between ADHD and bruxism, current evidence still has methodological limitations. Despite the fact that we did not restrict our search to children and adolescents, we found only one study (31) in adults. Even though it included an expressive sample size (over 11,000 participants), there is still much to be investigated about this association in distinct populations worldwide. Moreover, there remains a lack of standardization in the diagnosis of bruxism, with many studies failing to specify the type of bruxism analyzed (i.e., sleep or awake) and the method of diagnosis, which hinders subgroup analysis. Also, we did not find longitudinal studies in the literature that could clarify the temporal relationship between ADHD and bruxism.

Therefore, we recommend that future research be conducted using a longitudinal design. It is also important to adopt standardized methods for detecting bruxism to reduce bias and improve comparability across studies, and the use of more representative samples.

### 4.1 Strengths

This study is characterized by its rigorous methodology, which involves an exhaustive search across five databases, as well as gray literature sources, thereby augmenting the comprehensiveness and reliability of the findings. To maintain study integrity and mitigate selection bias, the review employed two blinded reviewers assisted by a third reviewer in the decision-making process.

Also, we have opted to include only studies in which the diagnosis of ADHD was based on any edition of the DSM (2, 13). The Manual published by the American Psychiatric Association has been used as a guide to diagnose and classify mental disorders, standardizing the diagnostic criteria and facilitating communication among healthcare professionals and researchers. The DSM (2, 13), criteria are used in research to identify groups of individuals with similar characteristics, and it is important when collecting and analyzing data (18). To restrict our eligibility criteria, we found more comparable studies.

## 5 Conclusion

Individuals with ADHD are two times more likely to have bruxism compared to controls.

### 5.1 Supporting information/data availability statement

#### 5.1.1 Protocol and registration

A systematic review protocol based on PRISMA-P (46, 47), was developed and registered in the International Prospective Register of Systematic Reviews (PROSPERO; ID: CRD42024538099). The protocol of this study was previously published in PLOS ONE (48). This review was reported in accordance with the PRISMA 2020 guidelines (46).

#### 5.1.2 Amendments to the registered protocol

Some deviations from the original protocol (PROSPERO ID: CRD42024538099) occurred during the review process. First, the exclusion criteria were refined to improve the specificity of included studies and ensure consistent diagnostic standards, particularly regarding ADHD diagnosis and comorbidities. Second, subgroup analyses were adjusted: while the protocol initially planned subgroups based on age (children, adolescents, or adults), type of bruxism (sleep/awake and diagnostic certainty), ADHD diagnosis method, and sampling strategy (convenience vs. population-based), only subgrouping by age group was performed. This decision was based on the availability and comparability of data across studies.

## Data Availability

The original contributions presented in the study are included in the article/**Supplementary Material**, further inquiries can be directed to the corresponding author/s.
