# Supplementary material for: Association between attention deficit hyperactivity disorder and bruxism: a systematic review
Source: Front Psychiatry. 2026 Sep 1;17:1762324. doi: 10.3389/fpsyt.2026.1762324 (PMC13576150; doi:10.3389/fpsyt.2026.1762324)
Supplement: Supplementary file 1 [file Table1.docx]

**Appendix A1 Search Strategies.**

| **Database** | **String (June 5, 2025)** |
| --- | --- |
| **Embase** | ('bruxism'/exp OR bruxism OR 'bruxomania'/exp OR bruxomania OR bruxisms OR bruxers OR bruxing OR bruxists OR bruxist OR bruxer OR 'sleep bruxism'/exp OR 'sleep bruxism' OR 'nocturnal bruxism'/exp OR 'nocturnal bruxism' OR 'sleep related bruxism'/exp OR 'sleep related bruxism' OR 'awake bruxism'/exp OR 'awake bruxism' OR 'bruxism during sleep'/exp OR 'bruxism during sleep' OR 'night-time bruxism'/exp OR 'night-time bruxism' OR 'nighttime bruxism'/exp OR 'nighttime bruxism' OR 'sleep-associated bruxism'/exp OR 'sleep-associated bruxism') AND ('attention deficit disorder with hyperactivity'/exp OR 'attention deficit disorder with hyperactivity' OR 'attention deficit disorders with hyperactivity' OR 'attention deficit hyperactivity disorder'/exp OR 'attention deficit hyperactivity disorder' OR 'attention deficit hyperactivity disorders' OR 'attention deficit disorder'/exp OR 'attention deficit disorder' OR 'attention deficit disorders' OR 'attention deficit' OR 'deficit-hyperactivity disorder' OR 'adhd' OR 'adhd'/exp OR 'adhd' OR 'hyperactivity'/exp OR hyperactivity) |
| **MEDLINE (via PubMed)** | "Bruxism"[MeSH Terms] OR "Bruxism"[All Fields] OR "Bruxomania"[All Fields] OR "bruxisms"[All Fields] OR "bruxers"[All Fields] OR "bruxing"[All Fields] OR "bruxists"[All Fields] OR "bruxist"[All Fields] OR "bruxer"[All Fields] OR "Sleep Bruxism"[MeSH Terms] OR "Sleep Bruxism"[All Fields] OR "awake bruxism"[All Fields] AND ("Attention Deficit Disorder with Hyperactivity"[MeSH Terms] OR "Attention Deficit Disorder with Hyperactivity"[All Fields] OR "Attention Deficit Disorders with Hyperactivity"[All Fields] OR "attention deficit hyperactivity disorder"[All Fields] OR "attention deficit hyperactivity disorders"[All Fields] OR "attention deficit hyperactivity disorder"[All Fields] OR "attention deficit hyperactivity disorders"[All Fields] OR "Attention Deficit Disorder"[All Fields] OR "Attention Deficit Disorders"[All Fields] OR "Attention Deficit"[All Fields] OR "Deficit-Hyperactivity Disorder"[All Fields] OR "ADDH"[Title/Abstract] OR "ADHD"[Title/Abstract] OR "Hyperactivity"[All Fields]) |
| **LILACS** | (bruxism OR bruxomania OR bruxisms OR bruxers OR bruxing OR bruxists OR bruxist OR bruxer OR "Sleep Bruxism" OR "awake bruxism" OR bruxismo OR "Bruxismo del Sueño") AND ("Attention Deficit Disorder with Hyperactivity" OR "Attention Deficit Disorders with Hyperactivity" OR "attention deficit hyperactivity disorder" OR "attention deficit hyperactivity disorders" OR "attention deficit hyperactivity disorder" OR "attention deficit hyperactivity disorders" OR "Attention Deficit Disorder" OR "Attention Deficit Disorders" OR "Hyperkinetic Syndrome" OR "Deficit-Hyperactivity Disorder" OR "ADDH" OR "ADHD" OR hyperactivity OR "Transtorno do Deficit de Atenção com Hiperatividade" OR "TDAH" OR "ADHD" OR "Síndrome Hipercinética" OR "Transtorno da Falta de Atenção" OR "Transtorno da Falta de Atenção com Hiperatividade" OR "Transtorno de Hiperatividade e Falta de Atenção" OR "Transtorno do Deficit de Atenção" OR "Trastorno por Déficit de Atención con Hiperactividad" OR "Perturbación de la Actividad y de la Atención" OR "Síndrome Hipercinético" OR "Trastorno Hipercinético con Déficit de la Atención" OR "Trastorno de Déficit de Atención con Hiperactividad" OR "Trastorno de Hiperactividad con Déficit de Atención" OR "Trastorno de Hiperactividad y Déficit de Atención" OR "Trastorno o Síndrome Deficitario de la Atención con Hiperactividad" OR "Trastorno por Déficit de Atención" OR "trastornos de déficit de atención con hiperactividad" OR "trastorno de déficit de atención" OR hiperatividade) AND ( db:("LILACS")) |
| **Livivo** | (Bruxism OR Bruxomania OR bruxisms OR bruxers OR bruxing OR bruxists OR bruxist OR bruxer OR "Sleep Bruxism" OR "awake bruxism") AND ("Attention Deficit Disorder with Hyperactivity" OR "Attention Deficit Disorders with Hyperactivity" OR "attention deficit hyperactivity disorder" OR "attention deficit hyperactivity disorders" OR "attention deficit hyperactivity disorder" OR "attention deficit hyperactivity disorders" OR "Attention Deficit Disorder" OR "Attention Deficit Disorders" OR ""attention deficit" OR "Deficit-Hyperactivity Disorder" OR "ADDH" OR "ADHD" OR Hyperactivity) |
| **Scopus** | TITLE-ABS-KEY(Bruxism OR Bruxomania OR bruxisms OR bruxers OR bruxing OR bruxists OR bruxist OR bruxer OR "Sleep Bruxism" OR "nocturnal bruxism" OR "sleep related bruxism" OR "awake bruxism") AND TITLE-ABS-KEY("Attention Deficit Disorder with Hyperactivity" OR "Attention Deficit Disorders with Hyperactivity" OR "attention deficit hyperactivity disorder" OR "attention deficit hyperactivity disorders" OR "attention deficit hyperactivity disorder" OR "attention deficit hyperactivity disorders" OR "Attention Deficit Disorder" OR "Attention Deficit Disorders" OR "Attention Deficit Disorder" OR "Deficit-Hyperactivity Disorder" OR "ADHD" OR "ADHD" OR Hyperactivity) |
| **Web of Science** | TS=(Bruxism OR Bruxomania OR bruxisms OR bruxers OR bruxing OR bruxists OR bruxist OR bruxer OR "Sleep Bruxism" OR "awake bruxism") AND TS=("Attention Deficit Disorder with Hyperactivity" OR "Attention Deficit Disorders with Hyperactivity" OR "attention deficit hyperactivity disorder" OR "attention deficit hyperactivity disorders" OR "attention deficit hyperactivity disorder" OR "attention deficit hyperactivity disorders" OR "Attention Deficit Disorder"OR "Attention Deficit Disorders" OR ""attention deficit" OR "Deficit-Hyperactivity Disorder" OR "ADHD" OR "ADHD" OR Hyperactivity) |
| **Google Scholar** | (Bruxism OR "Sleep Bruxism" OR "awake bruxism" OR Bruxismo OR "Bruxismo do Sono" OR OR "Bruxismo del Sueño") AND ("Attention Deficit Disorder with Hyperactivity" OR "Attention Deficit Disorder" OR "ADDH" OR "ADHD" OR Hyperactivity OR "Transtorno do Deficit de Atenção com Hiperatividade" OR "TDAH" OR "ADHD" OR Hiperatividade) |
| **ProQuest Dissertations & Theses Global (PQDT Global)** | noft(Bruxism OR Bruxomania OR bruxisms OR bruxers OR bruxing OR bruxists OR bruxist OR bruxer OR "Sleep Bruxism" OR "awake bruxism") AND noft("Attention Deficit Disorder with Hyperactivity" OR "Attention Deficit Disorders with Hyperactivity" OR "attention deficit hyperactivity disorder" OR "attention deficit hyperactivity disorders" OR "attention deficit hyperactivity disorder" OR "attention deficit hyperactivity disorders" OR "Attention Deficit Disorder" OR "Attention Deficit Disorders" OR ""attention deficit" OR "Deficit-Hyperactivity Disorder" OR "ADHD" OR "ADHD" OR Hyperactivity) |
