# Supplementary material for: Association between attention deficit hyperactivity disorder and bruxism: a systematic review
Source: Front Psychiatry. 2026 Sep 1;17:1762324. doi: 10.3389/fpsyt.2026.1762324 (PMC13576150; doi:10.3389/fpsyt.2026.1762324)
Supplement: Supplementary file 2 [file Table2.docx]

**Appendix 2** Articles Excluded and Reasons for Exclusion (n=53).

| 1. N | Author | Reason for Exclusion |
| --- | --- | --- |
| 1. | Adel et al 2021[^1^](https://www.zotero.org/google-docs/?ZmsRJ8) | 2 |
| 2. | Adler et al 2009 [^2^](https://www.zotero.org/google-docs/?jVRDum) | 2 |
| 3. | Armstrong et al 2014[^3^](https://www.zotero.org/google-docs/?LLtQjI) | 2 |
| 4. | Aykutlu et al 2018[^4^](https://www.zotero.org/google-docs/?LJZ6qt) | 3 |
| 5. | Bimstein et al 2008 [^5^](https://www.zotero.org/google-docs/?40UFkR) | 3 |
| 6. | Blumer et al 2022[^6^](https://www.zotero.org/google-docs/?NOhqUI) | 2 |
| 7. | Bruni et al 2021[^7^](https://www.zotero.org/google-docs/?s5dpLH) | 3 |
| 8. | Chavez Moreira 2023[^8^](https://www.zotero.org/google-docs/?bFwkOU) | 3 |
| 9. | Chin et al 2019[^9^](https://www.zotero.org/google-docs/?iL1Syn) | 2 |
| 10. | Ciopat et al 2013[^10^](https://www.zotero.org/google-docs/?n2wOtF) | 3 |
| 11. | da Paz Oliveira and Predella-Hallinan 2021[^11^](https://www.zotero.org/google-docs/?aeXizl) | 3 |
| 12. | Dashdorj et al 2010[^12^](https://www.zotero.org/google-docs/?zCdNN4) | 3 |
| 13. | de Matos and Veríssimo 2013[^13^](https://www.zotero.org/google-docs/?x3z7hA) | 2 |
| 14. | Delrosso and Ferri 2019[^14^](https://www.zotero.org/google-docs/?sz2Vpm) | 2 |
| 15. | Di Francesco et al 2004 [^15^](https://www.zotero.org/google-docs/?I67gZS) | 2 |
| 16. | Dogan 2017 [^16^](https://www.zotero.org/google-docs/?kp7xDF) | 3 |
| 17. | Farid et al 2010[^17^](https://www.zotero.org/google-docs/?keVbCZ) | 2 |
| 18. | Ghanizadeh 2008[^18^](https://www.zotero.org/google-docs/?81TrEc) | 2 |
| 19. | Guillén and Katherine 2021 [^19^](https://www.zotero.org/google-docs/?KYxYLD) | 3 |
| 20. | Hosiri et al 2018 [^20^](https://www.zotero.org/google-docs/?nbRZK2) | 4 |
| 21. | Huynh 2011 [^21^](https://www.zotero.org/google-docs/?llouwH) | 3 |
| 22. | Jesus et al 2013 [^22^](https://www.zotero.org/google-docs/?Y4VkAM) | 3 |
| 23. | Khajehpiri et al 2014 [^23^](https://www.zotero.org/google-docs/?89dkEu) | 2 |
| 24. | Kim et al 2010 [^24^](https://www.zotero.org/google-docs/?LXIXOc) | 3 |
| 25. | Lam et al 2011 [^25^](https://www.zotero.org/google-docs/?T2WkdU) | 2 |
| 26. | Machuca et al 2016 [^26^](https://www.zotero.org/google-docs/?VhpwNd) | 2 |
| 27. | Mahendran et al 2006 [^27^](https://www.zotero.org/google-docs/?c0swtO) | 2 |
| 28. | McElroy and Stoeckel 2023 [^28^](https://www.zotero.org/google-docs/?PXq5nb) | 2 |
| 29. | Mendhekar and Andrade 2008 [^29^](https://www.zotero.org/google-docs/?m6QGo9) | 3 |
| 30. | Mendhekar and Lohia 2009 [^30^](https://www.zotero.org/google-docs/?70lbpu) | 3 |
| 31. | Moreira and Belén 2023 [^8^](https://www.zotero.org/google-docs/?W7Nnwj) | 3 |
| 32. | Naguy et al 2019 [^31^](https://www.zotero.org/google-docs/?Ftry0T) | 3 |
| 33. | Ortiz 2022 [^32^](https://www.zotero.org/google-docs/?8lFs4Z) | 3 |
| 34. | Parchão and Gomes 2011 [^33^](https://www.zotero.org/google-docs/?DZzEmg) | 2 |
| 35. | Párraga et al 2007 [^34^](https://www.zotero.org/google-docs/?bQPN6j) | 3 |
| 36. | Razjouyan et al 2018 [^35^](https://www.zotero.org/google-docs/?Cdxzwt) | 2 |
| 37. | Reyad et al 2020 [^36^](https://www.zotero.org/google-docs/?mTScyS) | 3 |
| 38. | Riahai et al 2013 [^37^](https://www.zotero.org/google-docs/?rxHIe2) | 2 |
| 39. | Ribeiro-Lages et al 2023 [^38^](https://www.zotero.org/google-docs/?o0zyVq) | 2 |
| 40. | Ricketts et al 2022 [^39^](https://www.zotero.org/google-docs/?r7nfrU) | 2 |
| 41. | Sabater et al 2012 [^40^](https://www.zotero.org/google-docs/?m0A2zz) | 2 |
| 42. | Saglam and Akça 2017[^41^](https://www.zotero.org/google-docs/?9JUKhT) | 3 |
| 43. | Shur-Fen 2006 [^42^](https://www.zotero.org/google-docs/?YAHZor) | 2 |
| 44. | Silvestri et al 2005 [^43^](https://www.zotero.org/google-docs/?qzSIeL) | 3 |
| 45. | Silvestri et al 2009 [^44^](https://www.zotero.org/google-docs/?YOopJY) | 2 |
| 46. | Silvestri et al 2010 [^45^](https://www.zotero.org/google-docs/?4VYao7) | 3 |
| 47. | Soria-Bretones et al 2012 [^46^](https://www.zotero.org/google-docs/?ynFtzh) | 3 |
| 48. | Tecco and Tecco 2020 [^47^](https://www.zotero.org/google-docs/?YNm4KD) | 3 |
| 49. | Thasdelen et al 2015 [^48^](https://www.zotero.org/google-docs/?EkX4jW) | 3 |
| 50. | Tomás Vila et al 2008 [^49^](https://www.zotero.org/google-docs/?jzTCPD) | 2 |
| 51. | Vernallis 1995 [^50^](https://www.zotero.org/google-docs/?pIZl6d) | 2 |
| 52. | Vieira et al 2019 [^51^](https://www.zotero.org/google-docs/?ZwYeP0) | 3 |
| 53. | Yin et al 2022 [^52^](https://www.zotero.org/google-docs/?p2ZkgC) | 2 |

Exclusion Criteria:

Studies were excluded if they met any of the following criteria:

1. Involved specific populations, such as individuals with congenital anomalies; a history of bipolar disorder, psychosis, anxiety disorders, Tourette's disorder, autism; a history of substance abuse; or other neurological conditions associated with significant attention impairment;
2. Did not use any edition of the *Diagnostic and Statistical Manual of Mental Disorders* (DSM) for the diagnosis of ADHD;
3. Did not specify the method used to detect bruxism and/or ADHD; lacked data on bruxism; did not include a control group; or did not assess the association between bruxism and ADHD;
4. Were review articles, letters, editorials, books, conference abstracts, case reports, case series, opinion pieces, or clinical guidelines;
5. Full text was not available, even after three contact attempts with the corresponding authors over a three-week period.

**References:**

[1.](https://www.zotero.org/google-docs/?SGOYKE)	[Adel F. Almutairi et al., “Association of Oral Parafunctional Habits with Anxiety and the Big-Five Personality Traits in the Saudi Adult Population,” The Saudi Dental Journal 33, no. 2 (2021): 90–98, https://doi.org/10.1016/j.sdentj.2020.01.003.](https://www.zotero.org/google-docs/?SGOYKE)

[2.](https://www.zotero.org/google-docs/?SGOYKE)	[Lenard A. Adler et al., “Efficacy and Safety of OROS Methylphenidate in Adults With Attention-Deficit/Hyperactivity Disorder: A Randomized, Placebo-Controlled, Double-Blind, Parallel Group, Dose-Escalation Study,” Journal of Clinical Psychopharmacology 29, no. 3 (2009): 239–47, https://doi.org/10.1097/JCP.0b013e3181a390ce.](https://www.zotero.org/google-docs/?SGOYKE)

[3.](https://www.zotero.org/google-docs/?SGOYKE)	[Jeffrey M. Armstrong et al., “Associations of Child Insomnia, Sleep Movement, and Their Persistence With Mental Health Symptoms in Childhood and Adolescence,” Sleep 37, no. 5 (2014): 901–9, https://doi.org/10.5665/sleep.3656.](https://www.zotero.org/google-docs/?SGOYKE)

[4.](https://www.zotero.org/google-docs/?SGOYKE)	[Gürbüz HGA, Turhan L, Şendil N, et al. Oral Research Presentations. *Psychiatry Clin Psychopharmacol*. 2018;28(sup1):1-73. doi:10.1080/24750573.2018.1464273](https://www.zotero.org/google-docs/?SGOYKE)

[5.](https://www.zotero.org/google-docs/?SGOYKE)	[Bimstein E, Wilson J, Guelmann M, Primosch R. Oral characteristics of children with attention‐deficit hyperactivity disorder. *Spec Care Dentist*. 2008;28(3):107-110. doi:10.1111/j.1754-4505.2008.00021.x](https://www.zotero.org/google-docs/?SGOYKE)

[6.](https://www.zotero.org/google-docs/?SGOYKE)	[Blumer S, Eli I, Kaminsky-Kurtz S, Shreiber-Fridman Y, Dolev E, Emodi-Perlman A. Sleep-Related Breathing Disorders in Children—Red Flags in Pediatric Care. *J Clin Med*. 2022;11(19):5570. doi:10.3390/jcm11195570](https://www.zotero.org/google-docs/?SGOYKE)

[7.](https://www.zotero.org/google-docs/?SGOYKE)	[Bruni O, Giallonardo M, Sacco R, Ferri R, Melegari MG. The impact of lockdown on sleep patterns of children and adolescents with ADHD. *J Clin Sleep Med*. 2021;17(9):1759-1765. doi:10.5664/jcsm.9296](https://www.zotero.org/google-docs/?SGOYKE)

[8.](https://www.zotero.org/google-docs/?SGOYKE)	[Chávez Moreira, Ana Belén. Atención odontológica en niños con trastorno por déficit de atención e hiperactividad. Published online March 11, 2023.](https://www.zotero.org/google-docs/?SGOYKE)

[9.](https://www.zotero.org/google-docs/?SGOYKE)	[Chin WC, Huang YS, Chou YH, et al. Subjective and objective assessments of sleep problems in children with attention deficit/hyperactivity disorder and the effects of methylphenidate treatment. *Biomed J*. 2018;41(6):356-363. doi:10.1016/j.bj.2018.10.004](https://www.zotero.org/google-docs/?SGOYKE)

[10.](https://www.zotero.org/google-docs/?SGOYKE)	[Ciopat O, Díaz Román M, Urdanibia Centelles O, Rubio Sánchez P, Gomez Siurana E, Goyo Ibarra D. Sleep and ADHD. *Sleep Med*. 2013;14:e245-e246. doi:10.1016/j.sleep.2013.11.593](https://www.zotero.org/google-docs/?SGOYKE)

[11.](https://www.zotero.org/google-docs/?SGOYKE)	[Frange C, Coelho FMS, eds. *Sleep Medicine and Physical Therapy: A Comprehensive Guide for Practitioners*. Springer International Publishing; 2022. doi:10.1007/978-3-030-85074-6](https://www.zotero.org/google-docs/?SGOYKE)

[12.](https://www.zotero.org/google-docs/?SGOYKE)	[Abstracts of the Fourteenth International Congress of Parkinson’s Disease and *Movement* Disorders. *Mov Disord*. 2010;25(S2). doi:10.1002/mds.23162](https://www.zotero.org/google-docs/?SGOYKE)

[13.](https://www.zotero.org/google-docs/?SGOYKE)	[CAROLINA NORTON DE MATOS. O SONO E A PERTURBAÇÃO DE HIPERATIVIDADE E DÉFICE DE ATENÇÃO. Published online September 26, 2006.](https://www.zotero.org/google-docs/?SGOYKE)

[14.](https://www.zotero.org/google-docs/?SGOYKE)	[DelRosso LM, Ferri R. The prevalence of restless sleep disorder among a clinical sample of children and adolescents referred to a sleep centre. *J Sleep Res*. 2019;28(6):e12870. doi:10.1111/jsr.12870](https://www.zotero.org/google-docs/?SGOYKE)

[15.](https://www.zotero.org/google-docs/?SGOYKE)	[Lourdes M. DelRosso and Raffaele Ferri, “The Prevalence of Restless Sleep Disorder among a Clinical Sample of Children and Adolescents Referred to a Sleep Centre,” Journal of Sleep Research 28, no. 6 (2019): e12870, https://doi.org/10.1111/jsr.12870.](https://www.zotero.org/google-docs/?SGOYKE)

[16.](https://www.zotero.org/google-docs/?SGOYKE)	[Mine Elagoz Yuksel et al., “9th International Congress on Psychopharmacology & 5th International Symposium on Child and Adolescent Psychopharmacology,” Psychiatry and Clinical Psychopharmacology 27, no. sup1 (2017): 216–319, https://doi.org/10.1080/24750573.2017.1314599.](https://www.zotero.org/google-docs/?SGOYKE)

[17.](https://www.zotero.org/google-docs/?SGOYKE)	[Farid Zayeri. The Prevalence of Bruxism and Correlated Factors in Children Referred to Dental Schools of Tehran, Based on Parents’ Report. 2010;(Iran J Pediatr Jun 2010; Vol 20 (No 2), Pp:174-180).](https://www.zotero.org/google-docs/?SGOYKE)

[18.](https://www.zotero.org/google-docs/?SGOYKE)	[Ghanizadeh A. ADHD, bruxism and psychiatric disorders: does bruxism increase the chance of a comorbid psychiatric disorder in children with ADHD and their parents? Sleep Breath. 2008 Nov;12(4):375-80. doi: 10.1007/s11325-008-0183-9. Epub 2008 Apr 18. PMID: 18421490.](https://www.zotero.org/google-docs/?SGOYKE)

[19.](https://www.zotero.org/google-docs/?SGOYKE)	[Guzmán Guillén Millú Katherine, TUTOR:, Dr. Raúl Andrés Michalon Acosta, Guayaquil, octubre, 2021. FACTORES DE RIESGO DE BRUXISMO DE SUEÑO EN NIÑOS Y ADOLESCENTES.](https://www.zotero.org/google-docs/?SGOYKE)

[20.](https://www.zotero.org/google-docs/?SGOYKE)	[Hosiri T, Punyapas S, Sawangsri W. The prevalence and patterns of sleep problem in children with ADHD. [place unknown]: [publisher unknown]; [cited 2025 Sep 27]. Available from: https://mahidol.elsevierpure.com/en/publications/the-prevalence-and-patterns-of-sleep-problem-in-children-with-adh.](https://www.zotero.org/google-docs/?SGOYKE)

[21.](https://www.zotero.org/google-docs/?SGOYKE)	[“Presidential Lectures,” Sleep and Biological Rhythms 9, no. 4 (2011): 194–194, https://doi.org/10.1111/j.1479-8425.2011.00521.x.](https://www.zotero.org/google-docs/?SGOYKE)

[22.](https://www.zotero.org/google-docs/?SGOYKE)	[E. Jesus et al., “Sleep Habits and Sleep Disorders in Children with Attention Deficit-Hyperactivity Disorder. Influence of Treatment. A Multicenter Study,” Sleep Medicine 14 (December 2013): e117, https://doi.org/10.1016/j.sleep.2013.11.259.](https://www.zotero.org/google-docs/?SGOYKE)

[23.](https://www.zotero.org/google-docs/?SGOYKE)	[Khajehpiri Z, Mahmoudi-Gharaei J, Faghihi T, Karimzadeh I, Khalili H, Mohammadi M. Adverse reactions of Methylphenidate in children with attention deficit-hyperactivity disorder: Report from a referral center. J Res Pharm Pract. 2014 Oct;3(4):130-6. doi: 10.4103/2279-042X.145389. PMID: 25535621; PMCID: PMC4262859.](https://www.zotero.org/google-docs/?SGOYKE)

[24.](https://www.zotero.org/google-docs/?SGOYKE)	[Kim, J. Prevalence of sleep-disordered breathing symptoms in Korean children with attentiondeficit/hyperactivity disorder. 2010;Sleep-Volume 33, Issue 0, p. A328.](https://www.zotero.org/google-docs/?SGOYKE)

[25.](https://www.zotero.org/google-docs/?SGOYKE)	[Lam MH, Zhang J, Li AM, Wing YK. A community study of sleep bruxism in Hong Kong children: association with comorbid sleep disorders and neurobehavioral consequences. Sleep Med. 2011 Aug;12(7):641-5. doi: 10.1016/j.sleep.2010.11.013. PMID: 21684808.](https://www.zotero.org/google-docs/?SGOYKE)

[26.](https://www.zotero.org/google-docs/?SGOYKE)	[Valeria Carrión Machuca. RELACIÓN DE BRUXISMO DEL SUEÑO EN NIÑOS CON TRASTORNO DE DÉFICIT ATENCIONAL E HIPERACTIVIDAD POR OBSTRUCCIÓN DE VÍA AÉREA. Published online 2016.](https://www.zotero.org/google-docs/?SGOYKE)

[27.](https://www.zotero.org/google-docs/?SGOYKE)	[Mahendran R, Subramaniam M, Cai Y, Chan YH. Survey of sleep problems amongst Singapore children in a psychiatric setting. Soc Psychiatry Psychiatr Epidemiol. 2006 Aug;41(8):669-73. doi: 10.1007/s00127-006-0076-9. Epub 2006 May 26. PMID: 16732399.](https://www.zotero.org/google-docs/?SGOYKE)

[28.](https://www.zotero.org/google-docs/?SGOYKE)	[PREVALENCE OF BRUXISM AND ASSOCIATED RISK FACTORS IN CHILDREN AT SLU-CADE PEDIATRIC DENTAL RESIDENCY Clayton McElroy, D.M.D. A Thesis Presented to the Graduate Faculty of St. Louis University in Partial Fulfillment of the Requirements for the Degree of Master of Science in Dentistry.](https://www.zotero.org/google-docs/?SGOYKE)

[29.](https://www.zotero.org/google-docs/?SGOYKE)	[Dattatreya N. Mendhekar and C. Andrade, “Bruxism Arising During Monotherapy with Methylphenidate,” Journal of Child and Adolescent Psychopharmacology 18, no. 5 (2008): 537–38, https://doi.org/10.1089/cap.2008.18503.](https://www.zotero.org/google-docs/?SGOYKE)

[30.](https://www.zotero.org/google-docs/?SGOYKE)	[Dattatreya Mendhekar and Deep Lohia, “Worsening of Bruxism with Atomoxetine: A Case Report,” The World Journal of Biological Psychiatry 10, nos. 4–2 (2009): 671–72, https://doi.org/10.1080/15622970802576488.](https://www.zotero.org/google-docs/?SGOYKE)

[31.](https://www.zotero.org/google-docs/?SGOYKE)	[Naguy A, Elsori D, Alamiri B. Methylphenidate-Induced Nocturnal Bruxism Alleviated by Adjunctive Clonidine. J Child Adolesc Psychopharmacol. 2019 Feb;29(1):75-76. doi: 10.1089/cap.2018.0114. Epub 2018 Dec 21. PMID: 30575406.](https://www.zotero.org/google-docs/?SGOYKE)

[32.](https://www.zotero.org/google-docs/?SGOYKE)	[Tirado Ortiz, Ligia Isabel. Relación de los trastornos psicológicos con hábitos orales parafuncionales. 2022;Volume 0, Issue 0, pp.](https://www.zotero.org/google-docs/?SGOYKE)

[33.](https://www.zotero.org/google-docs/?SGOYKE)	[Parchão, Carla Maria Carvalho, Gomes, Ana Allen. Padrões de Sono em Crianças com Hiperactividade/Défice de Atenção: Estudo Numa Amostra Clínica. 2011;Volume 0, Issue 30641382, p. 46.](https://www.zotero.org/google-docs/?SGOYKE)

[34.](https://www.zotero.org/google-docs/?SGOYKE)	[Párraga, H. C., Párraga, M. I., Harris, D. K. Tic exacerbation and precipitation during atomoxetine treatment in two children with attention-deficit hyperactivity disorder. 2007;International Journal of Psychiatry in Medicine-Volume 37, Issue 4, pp. 415-424.](https://www.zotero.org/google-docs/?SGOYKE)

[35.](https://www.zotero.org/google-docs/?SGOYKE)	[Razjouyan, K., Danesh, A., Khademi, M., Davari-Ashtiani, R., Noorbakhsh, S. A comparative study of risperidone and aripiprazole in attention deficit hyperactivity disorder in children under six years old: A randomized double-blind study. 2018;Iranian Journal of Pediatrics-Volume 28, Issue 1, pp. 1-8. doi:10.5812/ijp.60087](https://www.zotero.org/google-docs/?SGOYKE)

[36.](https://www.zotero.org/google-docs/?SGOYKE)	[Ayman Antoun Reyad et al., “Bruxism and Psychotropic Medications,” Progress in Neurology and Psychiatry 24, no. 1 (2020): 31–35, https://doi.org/10.1002/pnp.560.](https://www.zotero.org/google-docs/?SGOYKE)

[37.](https://www.zotero.org/google-docs/?SGOYKE)	[Riahi F, Tashakori A, Izadi-Mazidi S, Salehi-Veysi M. Effectiveness of reboxetine in treatment of outpatient children and adolescents with attention deficit-hyperactivity disorder with comorbid anxiety disorders. Iran J Psychiatry. 2013 Oct;8(4):195-200. PMID: 25628714; PMCID: PMC4281655.](https://www.zotero.org/google-docs/?SGOYKE)

[38.](https://www.zotero.org/google-docs/?SGOYKE)	[Ribeiro-Lages MB, Dos Santos EC, Bendo CB, Fonseca-Gonçalves A, Serra-Negra JM, Maia LC. Association Between Attention-Deficit/Hyperactivity Disorder Symptoms and Sleep, Bruxism and Dental Trauma in Pediatric Population: A Preliminary Study. J Dent Child (Chic). 2023 Jan 15;90(1):22-30. PMID: 37106529.](https://www.zotero.org/google-docs/?SGOYKE)

[39.](https://www.zotero.org/google-docs/?SGOYKE)	[Ricketts EJ, Montalbano GE, Burgess HJ, McMakin DL, Coles ME, Piacentini J, Colwell CS. Sleep and chronotype in adults with persistent tic disorders. J Clin Psychol. 2022 Jul;78(7):1516-1539. doi: 10.1002/jclp.23323. Epub 2022 Feb 12. PMID: 35150595; PMCID: PMC9203900.](https://www.zotero.org/google-docs/?SGOYKE)

[40.](https://www.zotero.org/google-docs/?SGOYKE)	[MARTINEZ SABATER, A. et al. Sleep habits of the children in the Grau de Gandia: A descriptive study. Enferm. glob. [online]. 2012, vol.11, n.27, pp.124-141. ISSN 1695-6141.  https://dx.doi.org/10.4321/S1695-61412012000300008.](https://www.zotero.org/google-docs/?SGOYKE)

[41.](https://www.zotero.org/google-docs/?SGOYKE)	[Saǧlam, E., Akça, Ö F. Effectiveness of Daily Single Dose Buspirone Treatment in Sleep Bruxism. 2017;Psychiatry and Clinical Psychopharmacology-Volume 27, Issue 0, p. 166. doi:10.1080/24750573.2017.1308709](https://www.zotero.org/google-docs/?SGOYKE)

[42.](https://www.zotero.org/google-docs/?SGOYKE)	[Shur-Fen Gau S. Prevalence of sleep problems and their association with inattention/hyperactivity among children aged 6-15 in Taiwan. J Sleep Res. 2006 Dec;15(4):403-14. doi: 10.1111/j.1365-2869.2006.00552.x. Erratum in: J Sleep Res. 2010 Jun;19(2):379. Shur-Fen, Gau Susan [corrected to Shur-Fen Gau, Susan]. PMID: 17118097.](https://www.zotero.org/google-docs/?SGOYKE)

[43.](https://www.zotero.org/google-docs/?SGOYKE)	[Silvestri, R., Aricò, I., Gagliano, A., Raffaele, M., Calarese, T., Condurso, R., Calamoneri, F. Sleep disorders in ADHD children. *Proc WORLD Assoc SLEEP Med*. 2005;Volume 0, Issue 0, pp. 135-139.](https://www.zotero.org/google-docs/?SGOYKE)

[44.](https://www.zotero.org/google-docs/?SGOYKE)	[Silvestri, R., Gagliano, A., Aricò, I., Calarese, T., Cedro, C., Bruni, O., Condurso, R., Germanò, E., Gervasi, G., Siracusano, R., Vita, G., Bramanti, P. Sleep disorders in children with Attention-Deficit/Hyperactivity Disorder (ADHD) recorded overnight by video-polysomnography. *Sleep Med*. 2009;Volume 10, Issue 10, pp. 1132-8. doi:10.1016/j.sleep.2009.04.003](https://www.zotero.org/google-docs/?SGOYKE)

[45.](https://www.zotero.org/google-docs/?SGOYKE)	[Silvestri, R. Pediatric RLS: Diagnostic problems, co-morbidity and developmental impact in a Sicilian cohort. *Mov Disord*. 2010;Volume 25, Issue 0, p. S522. doi:10.1002/mds.23162](https://www.zotero.org/google-docs/?SGOYKE)

[46.](https://www.zotero.org/google-docs/?SGOYKE)	[Abstracts of the 21st Congress of the European Sleep Research Society. September 4-8, 2012. Paris, France. J Sleep Res. 2012 Sep;21 Suppl 1:1-387. doi: 10.1111/j.1365-2869.2012.01044.x. PMID: 22906134.](https://www.zotero.org/google-docs/?SGOYKE)

[47.](https://www.zotero.org/google-docs/?SGOYKE)	[Tecco, J. M., Tecco, S. Awake Bruxism Treated with Pregabaline in a Patient with Generalized Anxiety Disorder. *Psychiatr Danub*. 2020;Volume 32, Issue 0, pp. 33-35.](https://www.zotero.org/google-docs/?SGOYKE)

[48.](https://www.zotero.org/google-docs/?SGOYKE)	[Tasdelen, B. Sleep habits and related sleep disorders in children with attention deficit hyperactivity disorder. *Klin Psikofarmakol Bul*. Volume 25, Issue 0, pp. 86-87.](https://www.zotero.org/google-docs/?SGOYKE)

[49.](https://www.zotero.org/google-docs/?SGOYKE)	[Tomás Vila, M. Relación entre el trastorno por déficit de atención e hiperactividad y los trastornos del sueño. Resultados de un estudio epidemiológico en la población escolar de la ciudad de Gandía. *An Pediatr Barc Spain 2003*. 2008;Volume 69, Issue 3, pp. 251-257. http://www.ncbi.nlm.nih.gov/pubmed/18775271](https://www.zotero.org/google-docs/?SGOYKE)

[50.](https://www.zotero.org/google-docs/?SGOYKE)	[VERNALLIS FF. Teeth-grinding: some relationships to anxiety, hostility, and hyperactivity. J Clin Psychol. 1955 Oct;11(4):389-91. doi: 10.1002/1097-4679(195510)11:4<389::aid-jclp2270110413>3.0.co;2-f. PMID: 13252128.](https://www.zotero.org/google-docs/?SGOYKE)

[51.](https://www.zotero.org/google-docs/?SGOYKE)	[Vieira, S. P. Prevalence and factors associated to bruxism in dentistry students. *Sleep*. 2019;Volume 42, Issue 0, p. A91. doi:10.1093/sleep/zsz067.221](https://www.zotero.org/google-docs/?SGOYKE)

[52.](https://www.zotero.org/google-docs/?SGOYKE)	[Yin H, Yang D, Yang L, Wu G. Relationship between sleep disorders and attention-deficit-hyperactivity disorder in children. Front Pediatr. 2022 Jul 22;10:919572. doi: 10.3389/fped.2022.919572. PMID: 35935380; PMCID: PMC9354977.](https://www.zotero.org/google-docs/?SGOYKE)
