# Supplementary material for: Association between attention deficit hyperactivity disorder and bruxism: a systematic review
Source: Front Psychiatry. 2026 Sep 1;17:1762324. doi: 10.3389/fpsyt.2026.1762324 (PMC13576150; doi:10.3389/fpsyt.2026.1762324)
Supplement: Supplementary file 3 [file Table3.docx]

JBI Critical Appraisal Checklist for Systematic Reviews and Research

Syntheses

1. Is the review question clearly and explicitly stated?

2. Were the inclusion criteria appropriate for the review question?

3. Was the search strategy appropriate?

4. Were the sources and resources used to search for studies adequate?

5. Were the criteria for appraising studies appropriate?

6. Was critical appraisal conducted by two or more reviewers independently?

7. Were there methods to minimize errors in data

extraction?

8.Were the methods used to combine studies appropriate?

9. Was the likelihood of publication bias assessed?

10. Were recommendations for policy and/or practice supported by the reported data?

11. Were the specific directives for new research

appropriate?

Overall appraisal: Include □ Exclude □ Seek further info □

Comments (Including reason for exclusion)

**References:** https://jbi.global/sites/default/files/2019-05/JBI_Critical_Appraisal-Checklist_for_Systematic_Reviews2017_0.pdf
